# Supplementary material for: Learning from outcomes shapes reliance on moral rules versus cost–benefit reasoning
Source: Nat Hum Behav. 2025 Aug 11;10(2):268–87. doi: 10.1038/s41562-025-02271-w (PMC12932093; doi:10.1038/s41562-025-02271-w)
Supplement: Supplementary file 1 — Supplementary Methods and Results. [file 41562_2025_2271_MOESM1_ESM.pdf]

nature human behaviour

Supplementary information https://doi.org/10.1038/s41562-025-02271-w

Learning from outcomes shapes reliance on moral rules versus cost–benefit reasoning

In the format provided by the authors and unedited

Supplementary Information

S1 Supplementary Results

S1.1 Experiment 1

S1.1.1 Exploratory Analyses

Cognitive Reflection Test

The experimental manipulation had no significant effect on performance on the Cognitive Reflection Test, $t(384.65) = 0.44$, $p = .664$.

Awareness of Learning

A research assistant coded participants' open responses to a direct open question at the end of the study about whether they had changed "how they made decisions" during the study. The responses were coded as Yes/No/Not applicable (for irrelevant responses). The majority of our participants (79.2%) claimed that they had not changed their decision process from earlier dilemmas to later ones, even though our results suggest that most participants had exhibited some form of moral learning. The full qualitative responses are available in the online repository.

For the 158 participants reporting they did not experience moral learning in the CBR Success condition, we found significant effects of trial number on choices, $b = 0.14$, $z = 2.33$, $p = .020$, and ratings, $b = -1.71$, $t(1873.52) = -2.02$, $p = .043$. For the 149 participants reporting they did not experience moral learning in the Rule Success condition, we again found a significant effect on choices, $b = -0.17$, $z = -2.69$, $p = .007$ and no significant effect on moral judgments, $b = -1.18$, $t(1735.79) = -1.35$, $p = .178$. The effect on the OUS Sacrificial Harm Subscale was also significant for these participants across both conditions, $t(299.12) = 4.69$, $p < .001$, $d = 0.54$. Also see Experiment 2 for additional awareness results with continuous measures of awareness.

S1.2 Experiment 2

S1.2.1 Differences Between Conditions in Self-Report Measures of the Decision-Making Process

To measure additional potential effects of metacognitive learning, we used several self-report measures of (1) people's reliance on intuition versus deliberation and (2) people's reliance on rules versus CBR [85]. We expected participants in the CBR Success condition to report stronger reliance on CBR (vs. rules) and deliberation (vs. intuition) than participants in the Rule Success condition.

For CBR Action vignettes, we did not find significant differences between conditions in how much participants relied on CBR, $t(363.01) = 1.89, p = .118, d = 0.20$, one-sided; rules, $t(375.31) = 0.95, p = .345, d = 0.10$, one-sided; intuition, $t(374.56) = 0.45, p = .674, d = 0.05$, one-sided, or deliberation, $t(359.47) = 1.35, p = .269, d = 0.14$, one-sided (Holm-adjusted for the family of these four tests). Instead, participants in both conditions reported relying more on CBR than on rules, $t(379) = 9.99, p < .001, d = 0.51$, two-sided, and relying more on deliberation than

on intuition, $t(379) = 12.33, p < .001, d = 0.63$, two-sided. This is in line with the evidence for model-based learning in the other measures presented in the main text.

For Rule Action vignettes, we found significant differences between conditions in how much participants relied on CBR, $t(377.02) = 2.50, p = .026, d = 0.26$, one-sided, and deliberation, $t(370.45) = 1.80, p = .040, d = 0.19$. We did not find significant differences between conditions on how much they relied on rules, $t(372.78) = 2.22, p = .873, d = 0.23$, one-sided, and intuition, $t(367.59) = 1.14, p = .073, d = 0.12$, one-sided (Holm-adjusted for the family of these four tests). Participants in both conditions reported relying more on CBR than on rules, $t(379) = 4.50, p < .001, d = 0.23$, two-sided, and relying more on deliberation than on intuition, $t(379) = 13.36, p < .001, d = 0.69$, two-sided.

S1.2.2 Correlations Between Self-Report Scales

As shown in Table S1, we found a moderate positive correlation between deliberation and CBR and a moderate negative correlation between following rules and CBR. We also find a weak negative correlation between intuition and rules, and a weak positive correlation between intuition and CBR.

	Rules	Deliberation	Intuition
CBR	-0.42 (< .001)	0.48 (< .001)	0.15 (.003)
Rules		-0.03 (.620)	-0.18 (< .001)
Deliberation			0.01 (.920)

Table S1 Correlations Between Metacognitive Learning Scales in Experiment 2. Correlations are shown in the table as r values, and we include p-values from two-sided correlation tests in parentheses.

S1.2.3 Model-Based Learning (Including Descriptives and Visualization)

S1.2.4 Model-Free Learning

To measure model-free learning, we showed participants vignettes where they imagined being actors in a theatrical play carrying out harmful actions that break moral norms without causing negative outcomes, such as shooting someone with a prop gun (inspired by Cushman et al. [54] and Miller et al. [86]). They then rated their emotional response using a shortened version of PANAS-SF [87]. If model-free learning played a strong role, we would expect to see that evaluations of breaking norms would be more negative following the Rule Success condition (where breaking such norms had bad outcomes) than following the CBR Success condition (where breaking such norms had led to good outcomes). However, we did not find a significant difference between conditions, $t(377.08) = 0.62, p = .269, d = 0.06$, one-sided.

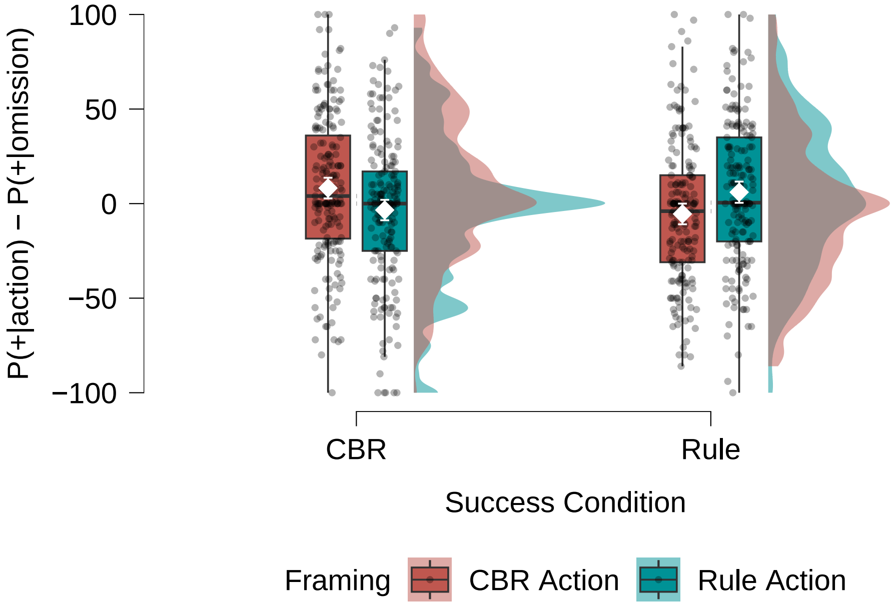

Figure S1 Ratings of the Probability of Good vs. Bad Outcomes Indicate Evidence for Metacognitive Model-Based Learning. Mean and 95% CIs are indicated in white ($N = 380$). Boxplots indicate median with IQR, and whiskers are constructed using 1.5 times the IQR.

S1.2.5 Relationship Between Estimates from the Model-Based Computational Model and Model-Based Behavioral Measure

To further corroborate the relationship between the modeling results and behavioral measures, we estimated the probability of good and bad outcomes for the different choice options (model-based measure) based on the posterior mean of the beta distribution and correlated these estimates to the ratings as given by participants. Specifically, we used a model-averaged estimate where the estimate from the model-based behavior models and the model-based metacognitive models were weighted based on the posterior probability of each of model. The estimates from the models are related to the probabilities indicated by participants for three out of four ratings, CBR Action: $r = .20$, $p < .001$, Rule Action: $r = .13$, $p = .008$, CBR Omission: $r = .11$, $p = .027$, Rule Omission: $r = 0.08$, $p = .112$.

S1.2.6 Model Predictions vs. Data

We performed Bayesian model averaging to compute a weighted average of the predictions of all six computational models. For each participant, each model's *a posteriori* prediction (fit) was weighted by the posterior probability that the participant's learning behavior was generated by that model. Figure S2 shows a comparison of the

resulting average predictions of the model ensemble to the empirical data. The fit indicates that the models can capture the qualitative phenomena and track the empirical data well.

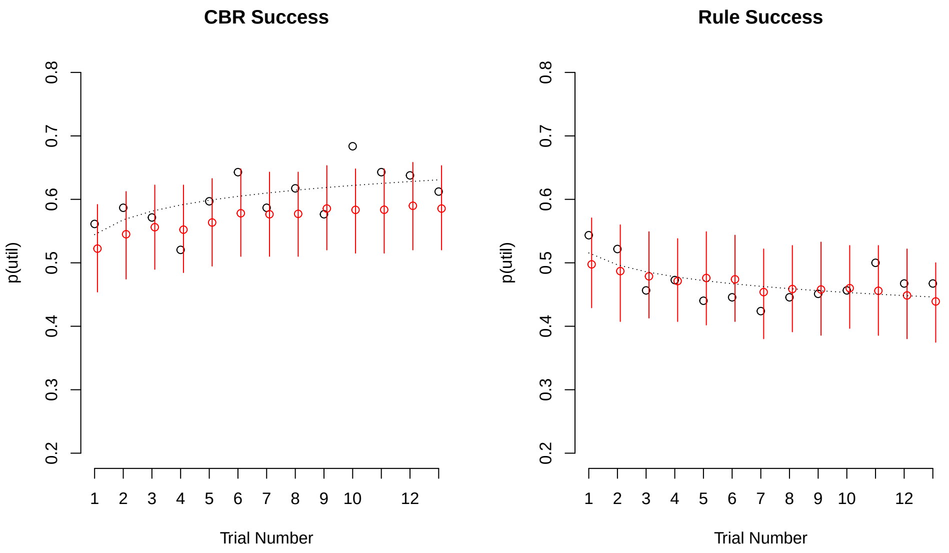

Figure S2 The Model Ensemble Represents Participants' Choice Data. The black dots indicate mean responses from participants and red dots indicate model predictions. Error bars indicate 95% prediction intervals.

S1.2.7 Non-Parametric Plot of Metacognitive Learning Interaction Effect

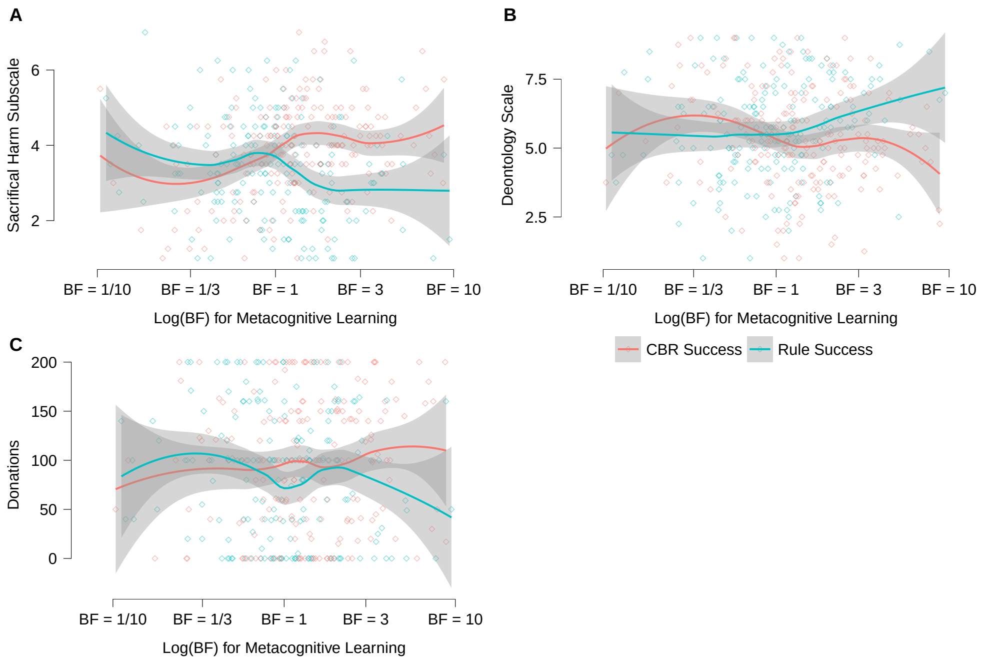

Figure S3 Local Regression Plot Visualising the Metacognitive Learning Interaction without Linearity Assumption. Panel A shows responses to the Sacrifical Harm Subscale from the Oxford Utilitarianism Scale; Panel B shows responses to the Deontology Subscale from the Deontological-Consequentialist Scale, and Panel C shows how much money participants donated to the charity supporting human challenge trials. Each panel compares responses between the CBR Success and Rule Success conditions as a function of the amount of evidence the participants' responses in the moral learning paradigm provided for metacognitive learning (BF). BF values of > 1 indicate evidence for metacognitive learning. For all panels, the confidence bands indicate 95% confidence level ($N = 380$). Note that there is less data to the edges of the plot and the estimates are therefore more variable.

S1.2.8 Determinants of Metacognitive Learning

Self-Report Responses of Metacognition

We used five questions to measure self-reported metacognitive learning (e.g., whether participants reported thinking about choosing to rely on rules vs. CBR). We find no significant relationship between these measures and evidence for metacognitive learning, $b = 0.0006, t(378) = -0.309, p = .758$.

Self-Report Responses of Strategy Integration

We asked participants three questions on whether they explicitly traded-off the benefits of the CBR option against the cost of breaking the rule. We also find no relationship

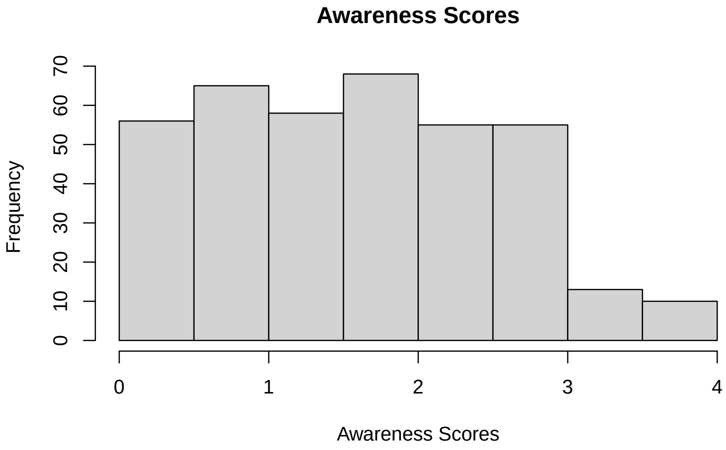

Figure S4 Distribution of Awareness Scores across Participants in Experiment 2.

between these measures and evidence for metacognitive learning, $b = 0.002, t(378) = -0.77, p = .443$.

Need for Cognition Scale

We find no significant relationship between need for cognition and metacognitive learning, $b = 0.07, t(374) = 1.27, p = .204$. Note that we excluded two participants from this analysis as their responses had one missing value.

Self-Reflection Scale

We also find no significant relationship between self-reflection and metacognitive learning, $b = 0.03, t(376) = 0.76, p = .451$.

Measures of Utilitarianism and Deontology

We find no significant relationship between the OUS Sacrificial Harm Subscale scores and metacognitive learning, $b = 0.025, t(378) = 0.74, p = .463$, and the DCS Deontology Subscale scores and metacognitive learning, $b = -0.015, t(378) = 0.58, p = .561$.

S1.2.9 Awareness of Learning

We computed a mean awareness score from the seven-item awareness measure (Cronbach's $\alpha = 0.88$). Figure S4 shows the distribution of scores across participants.

To test for the effect of choices when awareness is low, we add a covariate to the analysis of choices that is zero when participants have the lowest possible awareness. The results for all analyses from Experiment 2 when including this covariate are in the online repository https://osf.io/jcxft. In general, we find that there is usually no more effect of condition on the different dependent variables for participants with low

awareness; however, we also mostly do not see moderation by awareness. We believe this somewhat conflicting pattern may arise because including additional predictors in the model reduced the statistical power. Overall, we think these results are not conclusive enough to provide evidence for or against the role of awareness in moral learning.

S1.3 Experiment 3

S1.3.1 Computational Modeling Results Indicate Metacognitive Model-Based Learning (Confirmatory)

Our confirmatory replication of the cognitive modeling results in Experiment 2 found similar proportions of participants being best explained by each model (CBR Success: 86.97% model-based metacognitive; Rule Success: 56.17% model-based behavioral; see Table 1).

S1.3.2 Metacognitive Learning Transfers to a Range of Measures (Replication and Extension of Experiment 2)

Experiment 3 replicated the transfer effects on the OUS Sacrificial Harm Subscale, $t(829.06) = 7.50, p < .001, d = 0.52$, one-sided, and the DCS Deontology Subscale, $t(825.3) = 2.81, p = .003, d = 0.20$, one-sided.

Figure S5 visualizes the results of the donation task across all three vignettes. We find no difference between conditions, $t(832) = 1.55, p = .061$, one-sided. We find an effect of donation vignette, $F(2, 1664) = 53.76, p < .001$, and no significant interaction between condition and donation vignette, $F(2, 1664) = 1.82, p = .163$.

As preregistered, we tried to replicate the effect on donations in the "Human Challenge Trials" vignette we used in Experiment 2. Participants in the CBR Success condition allocated slightly more to the CBR option charity that supports human challenge trials ($M = 109.8$, 95% CI $[103.7, 115.9]$) compared to those in the Rule Success condition ($M = 103.7$, 95% CI $[97.6, 109.9]$), but this difference was not significant, $t(831.45) = 1.40, p = .082, d = 0.10$, one-sided.

In Experiment 3, we preregistered an analysis of transfer for metacognitive learners only. Figure S6 shows that learning from the consequences of moral decisions transferred to donation decisions for participants whose moral decisions provided strong evidence for metacognitive learning. Evidence for metacognitive learning moderated the effect of the experimental condition on all measures of transfer (OUS Sacrificial Harm Subscale, $b = 0.84, t(830) = 8.39, p < .001$; DCS Deontology Subscale, $b = -0.79, t(830) = 5.67, p < .001$; Donations, $b = 9.15, t(830) = 5.20, p < .001$; Donations only to support human challenge trials, $b = 19.38, t(830) = 3.72, p < .001$) and strong evidence of transfer for metacognitive learners on all measures of transfer (OUS Sacrificial Harm Subscale, $b = 2.48, t(830) = 10.62, p < .001$; DCS Deontology Scale, $b = -2.07, t(830) = 6.36, p < .001$; Donations, $b = 22.07, t(830) = 5.34, p < .001$, all one-sided).

We also observe a significant three-way interaction between type of donation, condition, and metacognitive learning. Therefore, we further test the interaction between donation decision and metacognitive learning for each donation vignette separately.

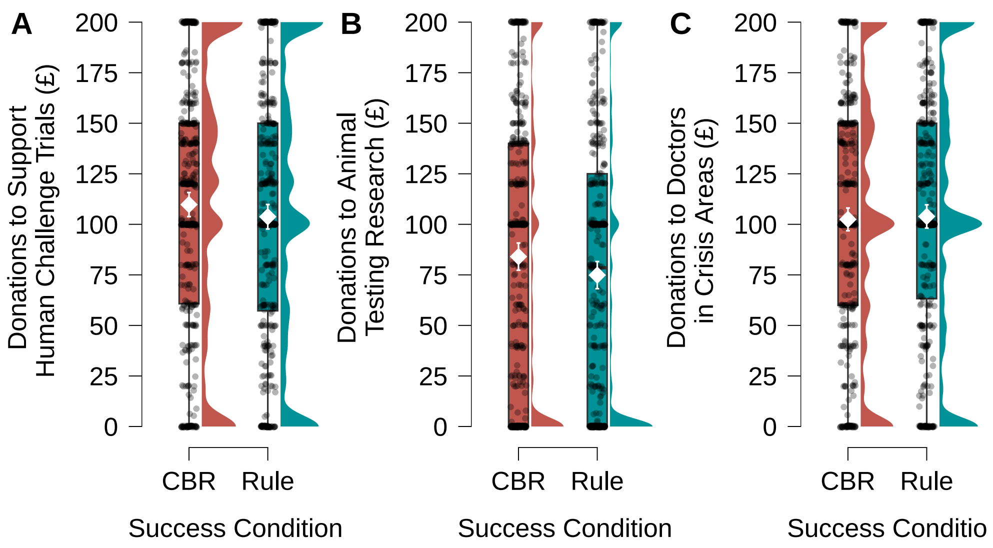

Figure S5 Donations to CBR Charities in Experiment 3. Panel A shows donations to 1Day Sooner, an organization advocating for human challenge trials to accelerate medical research (vs. the Medical Research Foundation, which funds more conventional medical research). Panel B shows donations to Breast Cancer Now, an organization that funds animal testing in research to combat breast cancer (vs. Breast Cancer UK, an organization that does not fund animal testing). Panel C shows donations to UK-Med, a charity that provides medical aid to conflict or disaster zones that have inherent risks (vs. Pathway, a charity that focuses on healthcare for homeless people in the UK, which is comparatively much less risky). More information about each charity is provided in the Methods section. Each panel compares responses between the CBR Success and Rule Success conditions as a function of the amount of evidence the participants' responses in the moral learning paradigm provided for metacognitive learning (BF). BF values of > 1 indicate evidence for metacognitive learning. Mean and 95% CIs are indicated in white. Boxplots indicate median with IQR, and whiskers are constructed using 1.5 times the IQR ($N = 380$).

This indicates strong moderation for the "Animal Testing" and "Human Challenge Trials" vignettes but not the "Doctors" vignette. For the main effect of condition, we also find a strong effect for the same two vignettes but not for "Doctors" ($b = 15.37, t(830) = 1.32, p = .188$).

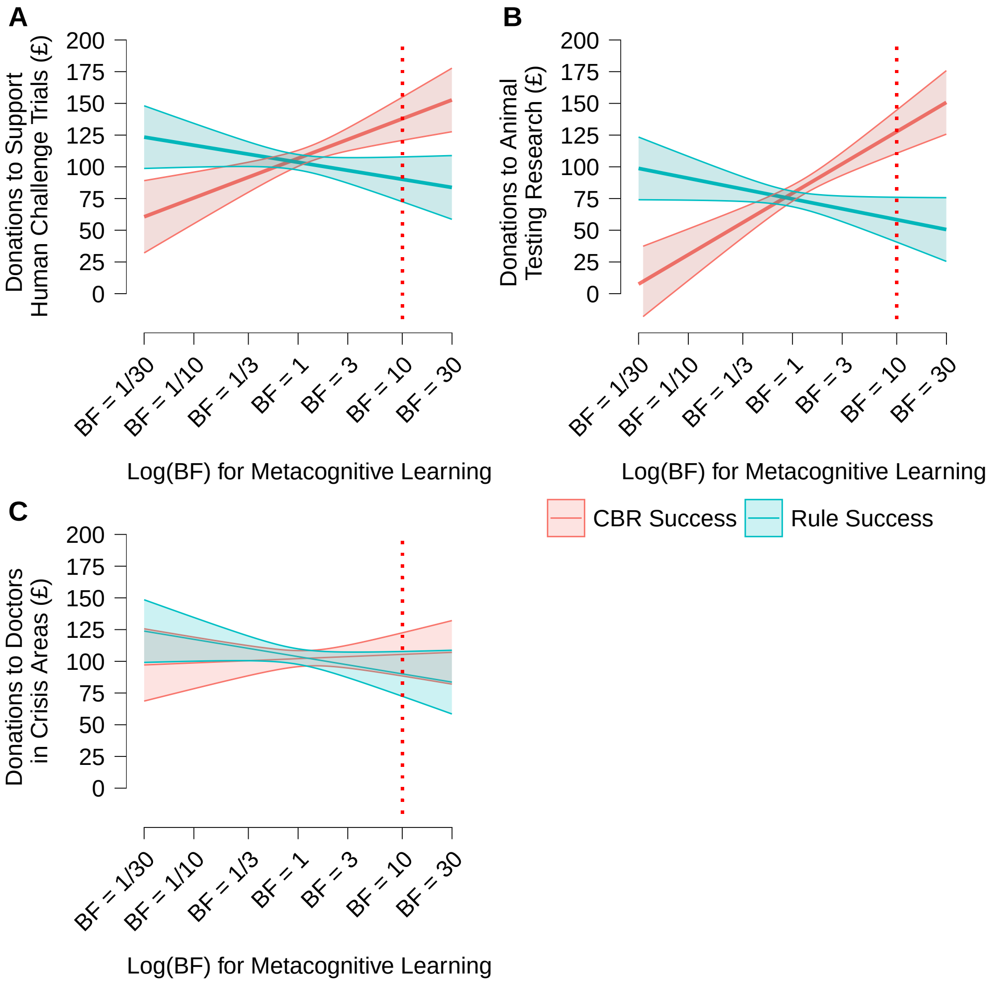

Figure S6 Donations to CBR Charities Are Moderated By Evidence for Metacognitve Learning. Panel A shows donations to 1Day Sooner, an organization advocating for human challenge trials to accelerate medical research (vs. the Medical Research Foundation, which funds more conventional medical research). Panel B shows donations to Breast Cancer Now, an organization that funds animal testing in research to combat breast cancer (vs. Breast Cancer UK, an organization that does not fund animal testing). Panel C shows donations to UK-Med, a charity that provides medical aid to conflict or disaster zones that have inherent risks (vs. Pathway, a charity that focuses on healthcare for homeless people in the UK, which is comparatively much less risky). More information about each charity is provided in the Methods section. Each panel compares responses between the CBR Success and Rule Success conditions as a function of the amount of evidence the participants' responses in the moral learning paradigm provided for metacognitive learning (BF). BF values of > 1 indicate evidence for metacognitive learning. The confidence bands indicate 95% CI.

S1.3.3 Non-Parametric Plot of Metacognitive Learning Interaction Effect

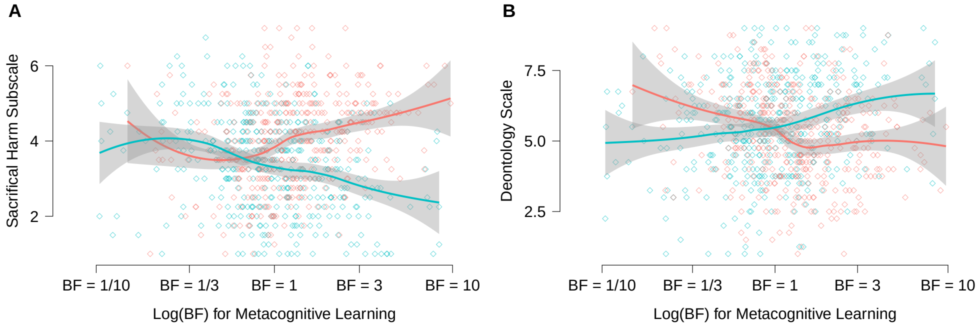

Figure S7 Local Regression Plot Visualising the Metacognitive Learning Interaction on OUS Sacrificial Harm Subscale (Panel A) and DCS Deontology Subscale without Linearity Assumption (Panel B) in Experiment 3. Each panel compares responses between the CBR Success and Rule Success conditions as a function of the amount of evidence the participants' responses in the moral learning paradigm provided for metacognitive learning (BF). BF values of > 1 indicate evidence for metacognitive learning. The confidence bands indicate 95% confidence level. Note that there is less data to the edges of the plot and the estimates are therefore more variable.

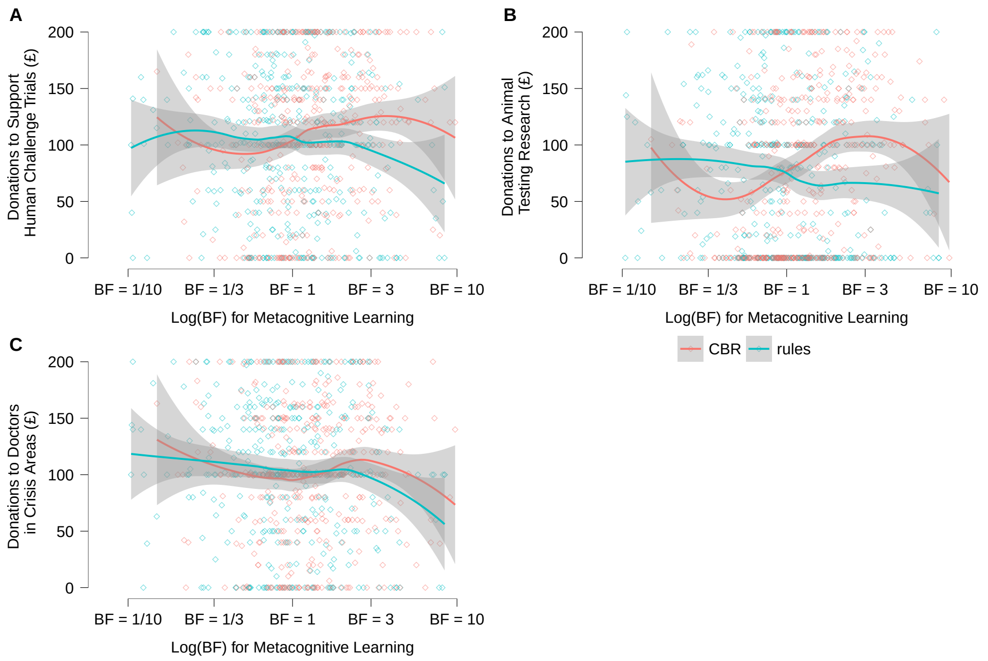

Figure S8 Local Regression Plot Visualizing the Metacognitive Learning Interaction on Donation Decisions without Linearity Assumption in Experiment 3. Panel A shows donations to human challenge trials, Panel B shows donations to breast cancer research that uses animal testing, and Panel C shows donations to doctors in crisis areas. More information about the donation task is provided in the Methods section. Each panel compares responses between the CBR Success and Rule Success conditions as a function of the amount of evidence the participants' responses in the moral learning paradigm provided for metacognitive learning (BF). BF values of > 1 indicate evidence for metacognitive learning. The confidence bands indicate 95% confidence level. Note that there is less data to the edges of the plot and the estimates are therefore more variable.

S1.3.4 Effect on Choices and Judgments (Replication of Experiments 1 and 2)

Experiment 3 replicated the effect of consequences of previous decisions on subsequent moral decisions we found in Experiments 1 and 2 when averaging across all participants. In particular, participants in the CBR Success condition became increasingly more likely to choose the CBR option with more experience, $b_{logtrialN} = 0.11, z = 2.77, p = .006$, one-sided, while participants in the Rule Success condition became increasingly less likely to choose the CBR option, $b_{logtrialN} = -0.20, z = -5.31, p < .001$, one-sided. As in Experiment 1, these effects were weaker for moral rightness judgments: we found no effect of trial number on the ratings in the CBR Success condition, $b = 0.04, t(4811.43) = 0.09, p = .930$, two-sided; however, we did find an effect in the Rule Success condition, $b = -1.45, t(2201.94) = 2.76, p = .012$, two-sided.

S1.3.5 Exploratory Analyses

Emotional Empathy and Empathic Concern Subscales

We find no significant relationship between emotional empathy and metacognitive learning, $b = -0.007, t(832) = 0.24, p = .808$, and no significant relationship between empathic concern and metacognitive learning, $b = 0.04, t(832) = 1.39, p = .166$.

Intelligence

We find no significant relationship between intelligence (as measured by a matrix reasoning task) and metacognitive learning, $b = -0.01, t(832) = 0.53, p = .597$.

Measures of Utilitarianism and Deontology

We find no significant relationship between the OUS Sacrificial Harm Subscale scores and metacognitive learning, $b = 0.011, t(832) = 0.49, p = .623$, and the DCS Deontology Subscale scores and metacognitive learning, $b = 0.010, t(832) = 0.60, p = .550$.

S1.4 Experiment 4

S1.4.1 Pilot Study to Test Whether Vignettes from Experiment 3 Were Viewed as Rules vs CBR Conflicts

In this pilot study, we presented participants with each donation vignette, then added two questions where we described a person who always made moral decisions by following deontological moral rules, and a person who always made moral decisions by "summing up the expected positive effects and subtracting negative effects". For each donation vignette, we asked which choice participants thought each person would prefer.

In particular, for "Doctor", 43 out of 102 participants indicated that it would be more in line with CBR to give to the homeless healthcare charity (which we had construed as the rule option), and 36 out of 102 participants indicated that it would coincide with following a moral rule to give to doctors in crisis situations, which we had construed as the CBR option. Therefore, the difference in the scenario in terms of perceived rules vs. CBR was only very small and smaller than for the other donation vignettes ("Animal Testing": 41 out of 102 people did not agree with our CBR option, but only 7 out of 102 did not agree with our rule option; "Human Challenge Trials": 28 out of 102 people did not agree with our CBR option, and 21 out of 102 with our rule option).

S1.4.2 Non-Parametric Plot of Metacognitive Learning Interaction Effect

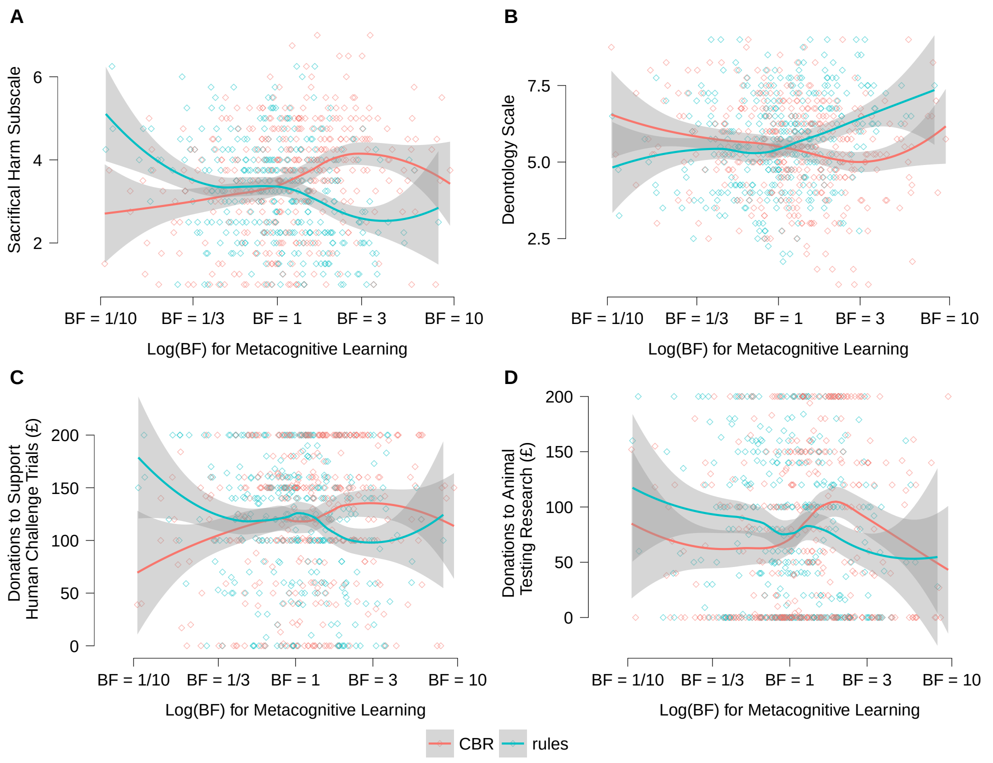

Figure S9 Local Regression Plot Visualizing the Metacognitive Learning Interaction without Linearity Assumption in Experiment 4. Panel A shows responses to the OUS Sacrificial Harm Subscale, Panel B shows responses to the DCS Deontology Subscale, Panel C shows donations to human challenge trials, and Panel D shows donations to breast cancer research that uses animal testing. More information about the donation task is provided in the Methods section. Each panel compares responses between the CBR Success and Rule Success conditions as a function of the amount of evidence the participants' responses in the moral learning paradigm provided for metacognitive learning (BF). BF values of > 1 indicate evidence for metacognitive learning. The confidence bands indicate 95% confidence level. Note that there is less data to the edges of the plot and the estimates are therefore more variable.

S1.4.3 Risk Aversion

We first compared the level of risk aversion between participants in the CBR Success and Rule Success conditions. This did not indicate a significant effect on either of the two risk aversion vignettes (Vignette 1: $b = -0.12, z = 0.76, p = .445$; Vignette 2: $b = 0.13, z = 0.82, p = .412$).

To ensure a fair comparison with the transfer results on donations, we further included an analysis of the effects on risk aversion for metacognitive learners. This indicated evidence for moderation of transfer to risky choices by evidence for metacognitive learning (Vignette 1: $b = -0.44, z = 2.46, p = 0.014$; Vignette 2: $b = -0.50, z = 2.68, p = .007$), and evidence for an effect on risk-taking for metacognitive learners (Vignette 1: $b = -0.87, z = 2.06, p = .039$; Vignette 2: $b = -1.31, z = 2.86, p = .004$).

Further, to test whether learning about risk aversion can account for the findings about the transfer of metacognitive learning, we test the transfer to donation decisions while controlling for risk aversion. This analysis still shows strong evidence for transfer from the learning to the donation task, $F(1, 721) = 16.07, p < .001$, and no evidence that risk aversion can predict donation behavior (Vignette 1: $F(1, 721) = 0.13, p = .718$; Vignette 2: $F(1, 721) = 0.06, p = .807$).

Overall, this indicates that while some participants may learn about risk aversion, learning about risk aversion cannot explain the transfer results to donation decisions or explain away meta-cognitive learning.

S1.4.4 Effect on Choices and Judgments (Replication of Experiments 1-3)

Experiment 4 replicated the effect of consequences of previous decisions on subsequent moral decisions we found in previous studies when averaging across all participants. In particular, participants in the CBR Success condition became increasingly more likely to choose the CBR option as the task progressed, $b_{logtrialN} = 0.11, z = 2.74, p = .006$, one-sided, while participants in the Rule Success condition became increasingly less likely to choose the CBR option, $b_{logtrialN} = -0.17, z = -4.29, p < .001$, one-sided. These effects were again weaker for moral rightness judgments: we found no effect of trial number on judgments in the CBR Success condition, $b = 0.82, t(4390.67) = 1.47, p = .142$, two-sided; however, we did find an effect in the Rule Success condition, $b = -1.33, t(1898.30) = 2.39, p = .017$, two-sided.

S1.4.5 Other Exploratory Measures

Relationship to Real World

Most participants agreed that the task gave them the opportunity to learn how to make better decisions in the real world ($M = 3.42$, with 50% of responses ≥ 4, indicating moderate or strong agreement). Those who agreed showed stronger evidence of metacognitive learning, $b = 0.09, t(725) = 2.97, p = .003$. Participants also agreed that the decisions, situations, and outcomes they encountered in the task were informative about the real world ($M = 3.77$, with 67% of responses ≥ 4). Agreement with this statement also predicted metacognitive learning, $b = 0.09, t(725) = 2.45, p = .015$.

Utility of Learning

Participants also indicated that the outcomes that occurred after their decisions were plausible ($M = 4.08$, with 83% of responses ≥ 4) and a good reflection of whether they made the right decision ($M = 3.65$, with 61% of responses ≥ 4); both these measures

predicted metacognitive learning and that making good decisions in these types of situations requires learning from experience ($M = 4.09$, with 80% of responses ≥ 4). All of these three questions also predicted evidence for metacognitive learning (plausibility, $b = 0.12, t(725) = 2.79, p = .005$; reflection of right decision, $b = 0.14, t(725) = 4.39, p < .001$; learning from experience, $b = 0.09, t(725) = 2.45, p = .015$). Additionally, participants disagreed that the outcomes were unrelated to their decision ($M = 2.07$, with 72% of responses ≤ 2) and were near the midpoint of the scale regarding whether to do the right thing and ignore the consequences ($M = 2.78$, with 41% of responses ≤ 2). However, these two items did not predict evidence for metacognitive learning ($b = -0.02, t(725) = 0.78, p = .438; b = -0.0007, t(725) = 0.025, p = .980$).

Engagement with Moral Decision-Making Task

We asked participants five questions related to task engagement and measured their agreement on a 5-point Likert scale. Participants reported taking the task seriously ($M = 4.76$), which predicted metacognitive learning, $b = 0.18, t(725) = 2.76, p = .006$. They also reported feeling quite good or bad after they saw good or bad outcomes ($M = 4.30$), which also predicted metacognitive learning, $b = 0.11, t(725) = 2.53, p = .012$. None of the other measures predicted metacognitive learning, including how vividly they imagined the scenarios ($M = 4.40$, $b = -0.02, t(725) = 0.35, p = .729$), and how important it was that they make the best possible decisions ($M = 4.58$, $b = -0.024, t(725) = 0.44, p = .661$).

Open-Minded Thinking About Evidence Scale

We found a significant relationship between open-minded thinking about evidence and metacognitive learning, $b = 0.09, t(725) = 2.00, p = .045$. However, due to the high number of statistical tests conducted and the high p-value, we would advice caution when interpreting this result.

Certainty of Knowledge Scale

We did not find a significant relationship between certainty of knowledge and metacognitive learning, $b = 0.01, t(725) = 1.76, p = .078$.

Measures of Utilitarianism and Deontology

We find no significant relationship between the OUS Sacrificial Harm Subscale scores and metacognitive learning, $b = 0.04, t(725) = 1.48, p = .141$, and the DCS Deontology Subscale scores and metacognitive learning, $b = -0.002, t(725) = 0.83, p = .934$.

Determinants of Learning Cannot Explain Differences Between Conditions

We did not find differences between conditions in how much participants perceived the task to be predictive of the real world, (the tasks helps to make better decisions in the real world, $t(723.35) = 1.34, p = .180$; the outcomes were informative about the real world, $t(720.22) = 1.37, p = .170$). Further, perceiving the outcomes as plausible did not differ between conditions, $t(719.84) = 1.42, p = .156$, and perceiving the outcomes

as a good reflection of whether one made the right decision did also not differ between conditions, $t(720.05) = 1.498, p = .135$. Taking the task seriously also did not differ between conditions, $t(723.51) = 0.03, p = .978$, and emotional engagement also did not differ between conditions, $t(724.08) = 1.37, p = .170$.

S1.4.6 Time Difference Between Learning and Transfer Tasks

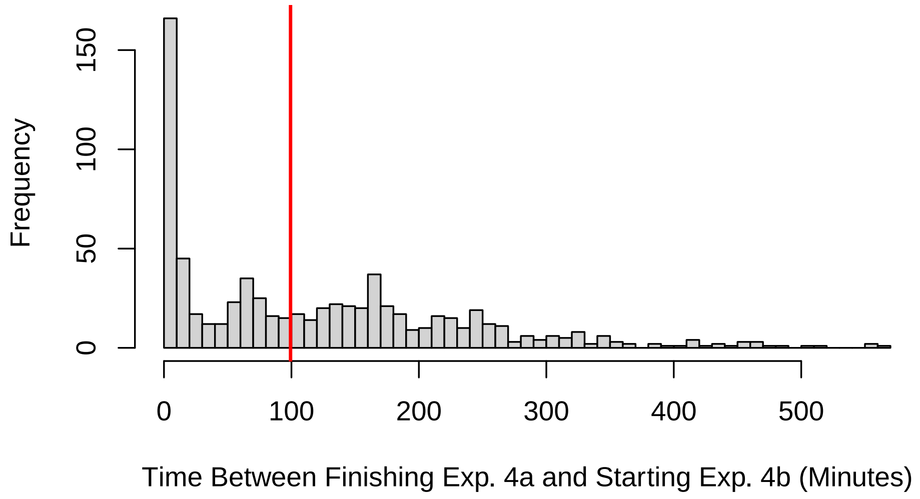

Figure S10 Time Difference Between the Learning Task and Transfer Measures in Experiment 4. The red line indicates the median time.

S1.5 Model Predictions vs. Data for Model-Based Learning Models (All Four Studies)

Figure S11 shows that our model of model-based metacognitive learning can capture the qualitative effects of learning in Experiments 1-4 for those participants whose behavior was best explained by this model. However, we also note that the model is conservative in comparison to the data. The reason is that as we zoom in on increasingly extreme participants (i.e., here, only the participants with the strongest metacognitive learning), the regularizing effect of the priors will shrink the model predictions towards 0.5. (This may be desirable model behavior from a predictive perspective, as we would expect the more extreme participants to be extreme to some also extent due to chance. Therefore, the shrinkage allows us to capture regression to the mean.)

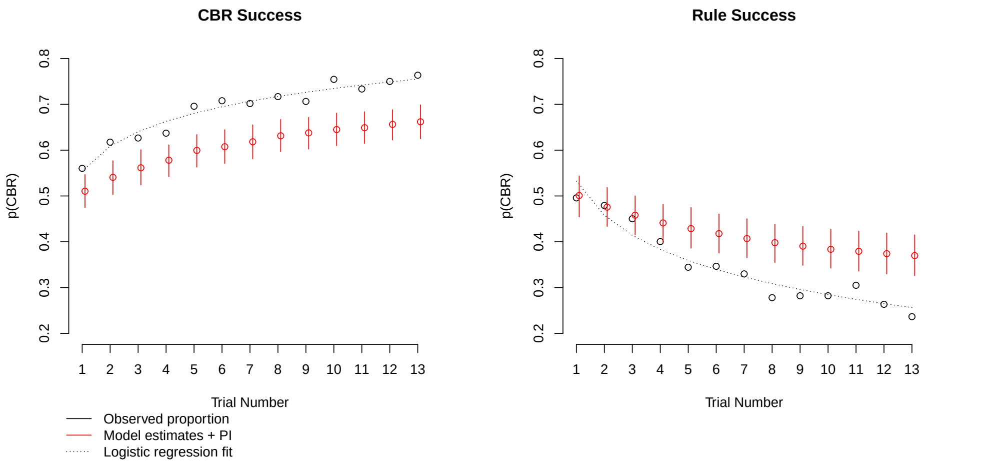

Figure S11 Fit of the Model-Based Metacognitive Learning Model to the Data from All Participants in Experiments 1-4 Whose Behavior was Best Explained By It. The black dots indicate the mean observed proportions of participants choosing the CBR option. All error bars indicate 95% prediction interval ($N = 2328$).

In addition to assessing the fit of the single-best model, we also inspected how well the predictions obtained by averaging the predictions of the two models of model-based learning fit the data from all participants. Figure S12 compares the predictions obtained by applying Bayesian model averaging to the predictions of the models of model-based learning to participants' learning curves. In both of these figures, the human data and the model predictions were averaged across Experiments 1-4.

Table S2 summarizes the results of a family-level inference on the proportion of participants whose learning behavior was best explained by one of the two models of model-based (metacognitive or behavioral) learning versus one of the two models of model-free (metacognitive or behavioral) learning. These findings should be taken with a grain of salt because the models also differ in one respect that is orthogonal to the distinction between model-based versus model-free RL.

S1.6 Cross-Study Analysis of Moral Rightness Judgments (All Four Studies)

We found that the interaction effect between trial number and framing on participants' moral rightness judgments were weaker than the effect on their decisions, reaching statistical significance in Experiment 1 and the Rule Success condition of Experiments 3 and 4, but not in Experiment 2 and the CBR Success condition of Experiments 3 and 4. To ascertain whether we see an overall effect on participants' moral judgments, we conducted an additional analysis of the effect on these judgment ratings across all experiments and conditions. We found a significant effect on the ratings, $F(1, 6144.66) = 16.17, p < .001$, and no evidence that this

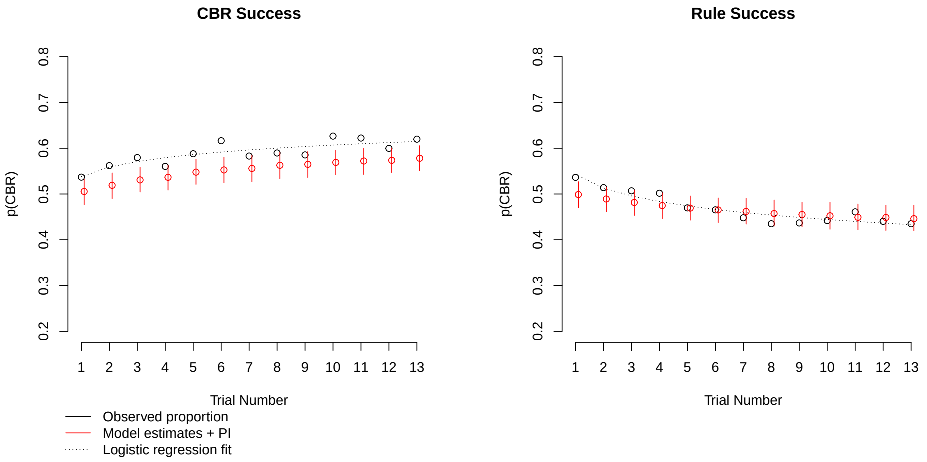

Figure S12 Comparisons of Predictions Obtained by Bayesian Model Averaging Across All Six Computational Models Against All Human Data from Experiments 1-4. The black dots indicate the mean observed proportions of participants choosing the CBR option. All error bars indicate 95% prediction interval ($N = 2328$).

Table S2 Expected Frequency ($\mathbb{E}[f|Y]$) of Participants Best Explained by Each Model Given the Data (Y) by Experiment and Experimental Condition.

	Expt 2				Expt 3				Expt 4			
	CBR		Rules		CBR		Rules		CBR		Rules	
	$\mathbb{E}[f|Y]$	φ	$\mathbb{E}[f|Y]$	φ	$\mathbb{E}[f|Y]$	φ	$\mathbb{E}[f|Y]$	φ	$\mathbb{E}[f|Y]$	φ	$\mathbb{E}[f|Y]$	φ
Model-Based Learning	86.56%	1	82.61%	1	93.35%	1	90.34%	1	86.46%	1	92.11%	1
Model-Free Learning	8.10%	0	12.66%	0	4.13%	0	6.34%	0	10.20%	0	3.22%	0
No Learning	5.35%	0	4.73%	0	2.53%	0	3.33%	0	3.34%	0	4.67%	0

Note. "CBR" denotes the CBR Success condition, and "Rule" denotes the Rule Success condition. The exceedance probability φ of a given model family is the probability that the proportion of participants best explained by a model from that family is greater than for any of the alternative model families.

effect is moderated by experiment ($F(3, 26708.45) = 0.85, p = .465$) or condition ($F(1, 26704.34) = 3.01, p = .083$).

S2 Supplementary Methods

S2.1 Experiment 1

S2.1.1 Data Analysis

To test the effect of trial number on choices, we preregistered to fit two logistic mixed effects models: one model using log trial number and one model using linear trial number. We preregistered to then select the one with lower BIC. As in the pre-specified analysis plan, we start from the maximal model and remove random effects, starting with the covariation of random intercepts and slopes and then continuing to random slopes (removing those random slopes with lower standard deviation first) until the model converges.

For the CBR choices in the CBR Success condition, this procedure led to a random effects model with main effects of *action framing* and *trial number* and independent random effect intercepts and trial number random slope. Note that because of the reduced sample size in the awareness of learning exploratory analysis (Section D in the main manuscript), which used only those participants unaware of the manipulation, we could only converge a model with random intercepts in that analysis. We report and visualize the model with *log trial number* for consistency, as the difference between the *log trial number* and *trial number* was minimal in results and BIC (3436.8 [nonlog] vs. 3435.1 [log]). For CBR choices in the Rule Success condition, the preregistered procedure led to a random effects model with main effects of *action framing* and *log trial number* and independent random effects for the intercept and the slope of the *log trial number*. Note that in the preregistration, we still used the typical moral psychology terminology and refer to the CBR option as utilitarian and the rule option as deontological.

For the moral judgments, we used the same approach to model selection between linear and log trial number and to reduce the random effects structure in the same way as for the choices. For the moral judgments in the CBR Success condition, the preregistered procedure led to a random effects models with with main effects for *appropriateness*, *log trial number*, *action framing*, the interaction of *log trial number* and *action framing*, and independent random effects for the intercept and the slope of *action framing*. Finally, for the moral judgments in the Rule Success condition, our preregistered procedure led to a random effects model with main effects of *appropriateness*, *log trial number*, *action framing*, the interaction of *log trial number* and *action framing*, as well as independent random effects for the intercept and the slopes of *trial number* and *action framing*.

S2.2 Experiment 2

S2.2.1 Metacognitive Learning

As an exploratory measure of people's metacognitive level learning, we asked participants to remember what they thought about when making their decisions, and how

well they thought each strategy would perform in similar moral dilemmas. Participants indicated their agreement to the following items on a scale of 0 ("Not at all") to 100 ("Entirely"):

- I remember thinking about how well relying on intuition tends to work in this kind of situation.
- I remember thinking about how well I can analyze the pros and cons in situations like this one.
- I remember asking myself if I can trust my gut feelings in situations like this one.
- I remember asking myself if I can trust my logical reasoning in situations like this one.
- I remember asking myself whether I should trust my head (logic) or my heart (intuitions and gut feelings).

S2.2.2 Awareness of Learning Measures

We first asked participants to think about which strategy (or strategies) they used when making decisions in the moral dilemmas and describe them in an open response box. Then, we asked them some questions about the strategies that they used. Participants indicated their agreement to the following items on a scale of 1 ("Not at all") to 5 ("Completely"):

- I remember using a different strategy in later moral dilemmas compared to earlier dilemmas.
- I am aware that the strategy I used to make decisions in the presented scenarios changed systematically over the course of this study.
- I made my decisions in the same way throughout the study.
- I am not aware that my strategy changed from the first scenario to the last one.
- The degree to which I relied on moral rules versus the action's anticipated consequences changed over the course of the experiment.
- I am aware that the degree to which I relied on my intuition changed systematically over the course of this study.
- I noticed becoming systematically more/less prone to base my decision on the action's anticipated consequences.

S2.2.3 Data Analysis

Choices and Judgments

We use the same analysis for choices and moral judgments as Experiment 1, with the difference that we preregistered to directly use log trial number as predictor (rather than using model comparison between log and linear trial number models).

Mixed Model for Model-Based Learning

We distinguish between two types of model-based learning: model-based learning about strategies (CBR vs. rules) and model-based learning about specific behaviors (action vs. omission). We describe how our analysis maps onto the different learning types in the preregistration (https://osf.io/7ds8a).

We specify the following mixed effects model testing the interaction of *condition* and *MB_type* on *MB_score*, and independent random effects for the intercept and the slopes for *MB_type*. *MB_score* refers to the difference between the probability of action achieving a good outcome minus the probability of an omission achieving a good outcome and *MB_type* indicates whether it was a rule action or a CBR action vignette. If the maximal model does not converge, we preregistered to remove the covariation of intercepts and slopes and then the random slopes. However, the mixed model with only random intercepts still displayed a boundary singular fit warning, we therefore also compared our analysis against within-subjects ANOVA, which lead to the same results.

S2.3 Experiment 3

S2.3.1 Testing Vignettes for the Donation Task

We ran two studies, Pilot 3A ($N = 42$) and Pilot 3B ($N = 40$), to test the donation task. In Pilot 3A, we gave participants three pairs of charities, namely 1Day Sooner vs. Medical Research Foundation, Breast Cancer Now vs. Breast Cancer UK, and 350.org (rule option) vs. Extinction Rebellion (CBR option). Figure S13 in the SI indicates that the last two vignettes were not well-balanced: participants preferred the rule option.

In Pilot 3B ($N = 40$), we revised the Breast Cancer Now vs. Breast Cancer UK vignette by adding more information about the importance of animal testing, and replaced the 350.org vs. Extinction Rebellion vignette with a choice between UK-Med vs. Pathway. Figure S14 shows that this set of vignettes was more well-balanced, hence we used these materials in the main experiment.

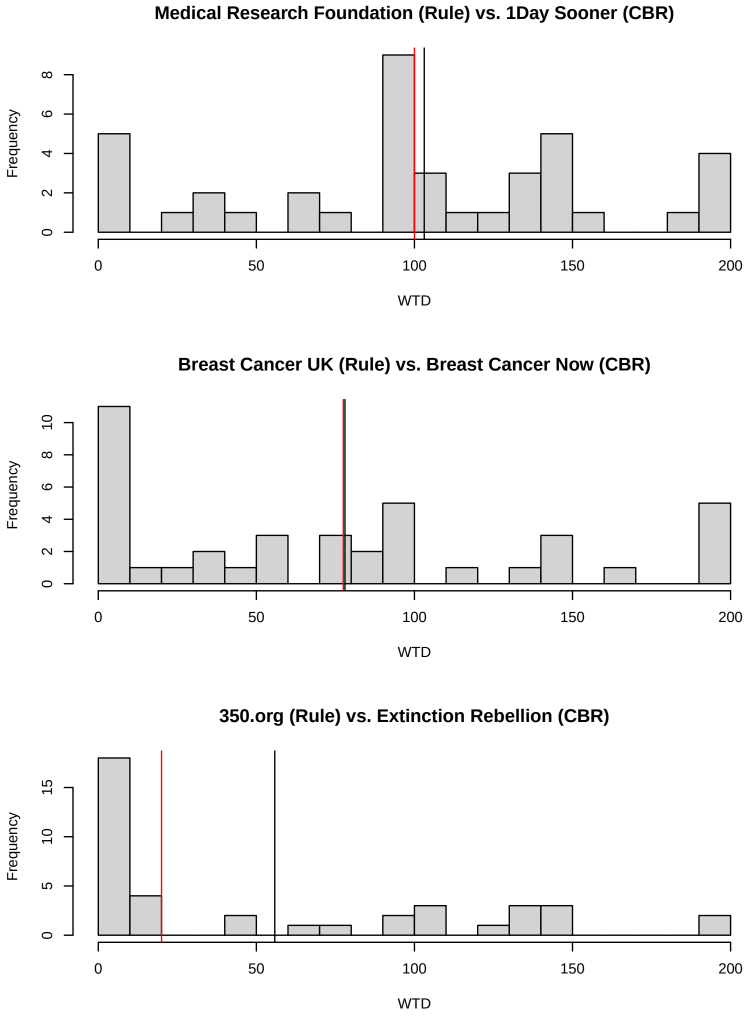

Figure S13 Histogram of Donation Results in Pilot 3A. Note that WTD indicates participants' willingness to donate to the CBR charity option. The black line indicates the mean and the red line indicates the median.

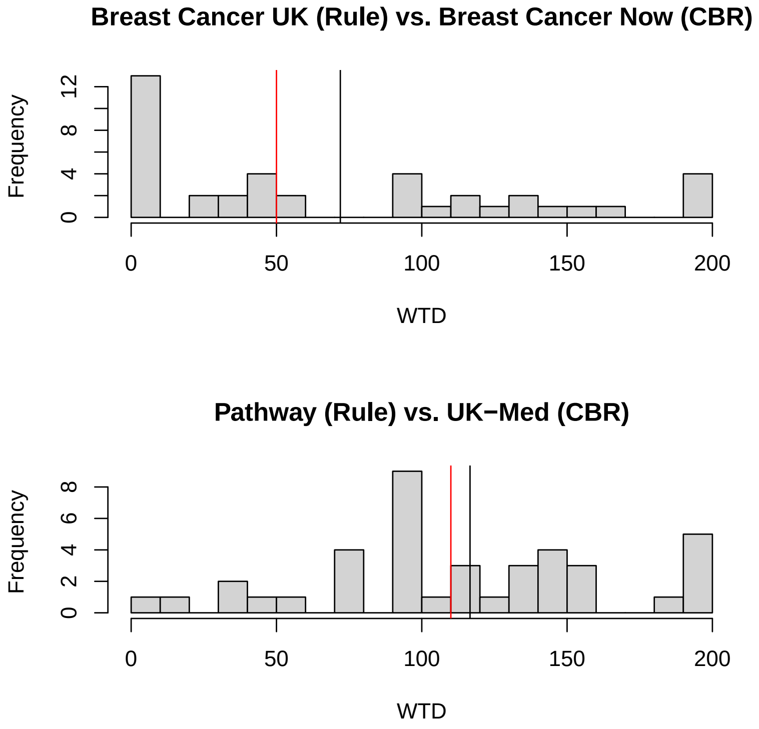

Figure S14 Histogram of Donation Results in Pilot 3B. Note that WTD indicates participants' willingness to donate to the CBR charity option. The black line indicates the mean and the red line indicates the median.

S2.4 Experiment 4

S2.4.1 Risk-Taking Vignette

The full text for the "Firefighter" vignettes is included below. Participants saw both vignettes in randomized order.

> *Imagine you are an experienced firefighter. You have just arrived at a scene where two buildings are on fire. A police officer at the scene informs you that there are people trapped inside each building. Although other firefighters are on their way, you know that the people inside the building cannot wait, and you and your team need to act now. As the leader of your team, you have to make critical decisions about who to save and who to let die.*
>
> ***Vignette 1***
>
> *In Building A, you see eight people inside a room. There is only one route that they can use to escape. They can climb out of the window and navigate to the ground floor by climbing down the building's exterior. Your experience tells you that there is a 25% chance that all eight people will survive this escape. However, there is a 75% chance that they won't make it and all eight of them will die.*

In Building B, you see that three people are trapped and their escape is completely cut off by flames. Based on your experience, you know with certainty that there is no way they can escape without your help. You only have the time and resources to save people in one building.

In summary, if you save the people in Building A, there is 100% certainty that three people in Building B will die. If you save the people in Building B, there is a 75% chance that the eight people in Building A will die.

Which do you prefer? (1) Save the eight people in Building A (100% certainty that the three people in Building B will die); (2) Save the three people in Building B (75% chance that the eight people in Building A will die)

How morally right is it to save the eight people in Building A (with 100% certainty that the three people in Building B will die)? (0 = "Not at all morally right", 100 = "Completely morally right")

Vignette 2

In Building O, you see that five people are trapped and their escape is completely cut off by flames. Based on your experience, you know with certainty that there is no way that they can escape without your help.

In Building P, you see 20 people inside a room. There is only one route that they can use to escape. They can climb out of the window and navigate to the ground floor by climbing down the building's exterior. Your experience tells you that there is a 50% chance that all 20 people will survive in the escape. However, there is a 50% chance that none of them will make it and all 20 of them will die. You only have the time and resources to save people in one building.

In summary, if you save the people in Building O, there is a 50% chance that the 20 people in Building P will die. If you save the people in Building P, there is 100% certainty that five people in Building O will die.

Which do you prefer? (1) Save the five people in Building O (50% chance that the 20 people in Building P will die); (2) Save the 20 people in Building P (100% certainty that five people in Building O will die)

How morally right is it to save the five people in Building O (with 50% chance that the 20 people in Building P will die)? (0 = "Not at all morally right", 100 = "Completely morally right")

S2.4.2 Perceived Utility of Learning Measures

After the learning paradigm, we showed participants some items on perceived utility of learning from outcomes and how much they perceived the scenarios to be informative for the real world. They indicated their agreement to these items on a scale of 1 ("Strongly Disagree") to 5 ("Strongly Agree").

- The outcomes that were reported after my decision were a good reflection of whether I made the right decision.
- The outcomes that were reported after my decision were plausible.
- The outcomes that were reported after my decision were random and unrelated to my decision. (R)
- This is the kind of situation where one should do the right thing and then ignore whatever happens afterwards. (R)

- Making good decisions in such complex situations requires learning from experience.
- This task gave me the opportunity to learn how to make better decisions in the real world.
- The decision situations and outcomes I encountered in this task were informative about the real world.

S2.4.3 Engagement with Moral Decision-Making Task

Directly after the learning paradigm, we asked participants the following questions. They were encouraged to answer as honestly as possible as their answer would not affect their pay, and indicated their agreement to these items on a scale of 1 ("Strongly Disagree") to 5 ("Strongly Agree").

- I imagined the scenarios very vividly when I made decisions.
- I took the decision-making task very seriously.
- I felt good when my decisions in this task turned out well.
- I felt bad when my decisions in this task turned out badly.
- It was important to me to make the best possible decisions.
